# Comparative genome analysis of PHB gene family reveals deep evolutionary origins and diverse gene function

**DOI:** 10.1186/1471-2105-11-S6-S22

**Published:** 2010-10-07

**Authors:** Chao Di, Wenying Xu, Zhen Su, Joshua S Yuan

**Affiliations:** 1 College of Biological Sciences, China Agricultural University, Beijing, China; 2 Department of Plant Pathology and Microbiology, Texas A&M University, College Station, TX 77843, USA; 3 Institute for Plant Genomics and Biotechnology, Texas A&M University, College Station, TX 77843, USA; 4 Institute for Genetics and Development, Chinese Academy of Sciences, Bejing, China

## Abstract

**Background:**

PHB (Prohibitin) gene family is involved in a variety of functions important for different biological processes. PHB genes are ubiquitously present in divergent species from prokaryotes to eukaryotes. Human PHB genes have been found to be associated with various diseases. Recent studies by our group and others have shown diverse function of PHB genes in plants for development, senescence, defence, and others. Despite the importance of the PHB gene family, no comprehensive gene family analysis has been carried to evaluate the relatedness of PHB genes across different species. In order to better guide the gene function analysis and understand the evolution of the PHB gene family, we therefore carried out the comparative genome analysis of the PHB genes across different kingdoms.

**Results:**

The relatedness, motif distribution, and intron/exon distribution all indicated that PHB genes is a relatively conserved gene family. The PHB genes can be classified into 5 classes and each class have a very deep evolutionary origin. The PHB genes within the class maintained the same motif patterns during the evolution. With* Arabidopsis* as the model species, we found that PHB gene intron/exon structure and domains are also conserved during the evolution. Despite being a conserved gene family, various gene duplication events led to the expansion of the PHB genes. Both segmental and tandem gene duplication were involved in Arabidopsis PHB gene family expansion. However, segmental duplication is predominant in Arabidopsis. Moreover, most of the duplicated genes experienced neofunctionalization. The results highlighted that PHB genes might be involved in important functions so that the duplicated genes are under the evolutionary pressure to derive new function.

**Conclusion:**

PHB gene family is a conserved gene family and accounts for diverse but important biological functions based on the similar molecular mechanisms. The highly diverse biological function indicated that more research needs to be carried out to dissect the PHB gene function. The conserved gene evolution indicated that the study in the model species can be translated to human and mammalian studies.

## Background

Prohibitin (PHB) is also known as band_7 domain proteins or SPFH (stomatins, prohibitins, flotillins and HflK/C) domain-containing proteins. PHB genes widely exist in a broad spectrum of species ranging from prokaryotes to eukaryotes [1-4]. Depending on the subcellular localization and other factors, PHB genes could be involved in important but diverse biological functions [5]. In human, PHB genes were found to be associated with the breast cancer phenotype, where PHB localizes in the nucleus of some breast cancer cell lines as a transcriptional regulator interacting with E2F, P53, and retinoblastoma (Rb) to regulate the expression of downstream genes. PHB gene can therefore serve as a tumour suppressor regulating cell-cycle progression and apoptosis [6-9]. Besides cell nucleus, PHBs were also found in lipid raft, an important component of cell membrane [1,2,4,6-11]. The plasma membrane PHBs were believed to serve as a target for small molecules in the inflammatory responses and to regulate the iron channels and membrane receptor [12-14]. Overall, PHB genes play important roles for various biological processes and are associated with different disease phenotypes.

Despite the diverse biological functions, most of molecular level studies for PHB genes were focused on their roles in mitochondria. In yeast and mammalian cells, PHB1 and PHB2 are highly homologous subunits that can interact with each other as a complex [15-17]. The assembled complex with 12 to 16 heterodimers is anchored to the mitochondrial inner membrane to play potentially diverse functions as indicated in various publications. PHB complex could interact with m-AAA protease to regulate the degradation of membrane proteins in mitochondria [18]. The PHB complex could also interact with stomatin-like protein (SLP-2/Stoml2) to regulate the stability of the components of respiratory chain complexes [17,19,20]. PHB proteins have also been proposed to directly or indirectly interact with mtDNA to regulate the oxidative phosphorylation (OXPHOS) system and reactive oxygen species (ROS) formation, which could lead to senescence phenotype in plants and* C.elegans *[21-24]. In additions, PHBs might be involved in maintaining crista morphology to recruit proteins into the inner membrane [25,26]. Overall, all of the aforementioned molecular studies suggested the regulatory function of PHB genes for cell proliferation [5,27].

Despite the progresses in the function studies, our understanding of the gene family is still rather limited. First, the function at both molecular and pathway level needs to be better defined. Different mechanisms for gene function have been proposed, but few were thoroughly defined for linking the molecular function with biological function. Second, many members of PHB gene family were not well studied in any given species. The diverse gene expression pattern as shown in the article indicated that the member of PHB gene family could account for very diverse functions. Third, despite the previous analyses of PHB gene function in yeast, mammalian cell, and C. elegans, very few studies have been carried out in plants and prokaryotes. Recent studies indicated that PHB genes may be involved in sensence phenotype and we have also discovered the potential function of some PHB genes in growth and defense processes. In order to lay the grounds for studying PHB gene function in different species, we therefore carried out the comparative gene family analysis of this important gene family to study the evolution-functional relevance of the family.

We therefore carried out a comparative gene family analysis of PHB genes from representative species in different biological kingdoms. Phylogenetic analysis of PHB genes from different kingdoms indicated the deep evolutionary roots of the PHB genes. Horizontal gene transfer between higher and lower species has also been found. The phylogenetic analysis is further confirmed by motif pattern, intron-exon structure, and domain distribution of the gene family that PHB genes within each class generally had conserved gene structure. We then focused on the gene duplication and expression analysis in model plant species* Arabidopsis.* Segmental duplication is the predominant for PHB genes, which confirms that the gene family is relatively conserved. Expression pattern analysis indicated that PHB genes could involved in a variety of functions ranging from development to abiotic and biotic stress responses. Using expression pattern as an indicator for gene function, we found that paralogs often evolve different functions during the evolution. Overall, PHB gene family is a relatively conserved gene family with rather diverse gene function. The evolution-function relationship indicated that the functional study in one species could provide meaningful information for another species. The study of PHB gene functions in model species could also help to elucidate the PHB gene function in cancer, aging, immunity, and others.

## Results

### Evolutionary relatedness of PHB genes in species from different biological kingdoms

In order to carry out a comprehensive analysis of PHB gene families across different species, we first identified the PHB genes from three major biological databases. The EMBL-EBI InterPro database contains 6476 proteins with a ‘IPR001107 Band7’ domain. The Pfam database contains 6416 proteins with the domain name of ‘*Band_7* (PF01145)’. The SMART (Simple Modular Architecture Research Tool) database contains 5913 proteins with the domain named as “SM00244 PHB”. As aforementioned, Band_7domain, SPFH domain and prohibitin domain all refers to the similar protein domain with different names. To be consistent, we therefore use the name prohibitin (PHB) proteins in this article [3]. PHB genes were widely distributed among most species, including many kinds of prokaryotes and eukaryotes. In order to study the evolution-function relationship of PHB genes, representative species from the kingdoms of* monera, fungi, plantae,* and* animalia* were chosen for phylogenetic analysis. Species names and their classification are as shown in Table 1.

**Table 1:** The species selection for PHB gene family analysis.

**Species**	**Logogram**	**ID Kingdom**	**Low–rank**	**Gene**	**number Sequence data source**
Arabidopsis thaliana	AT	Plantae	Dicot	17	http://www.arabidopsis.org/
Oryza sativa L.(rice)	LOC_OS	Plantae	Monocot	19	http://rice.plantbiology.msu.edu/
Medicago truncatula	MEDTR	Plantae	Dicot	7	http://www.medicago.org/
Populus tremula	POPTR	Plantae	Dicot	18	http://genome.jgi-psf.org
Sorghum bicolor	SORBI	Plantae	Monocot	11	http://genome.jgi-psf.org
Physcomitrella patens (moss)	PHYPA	Plantae	Bryophyte	16	http://genome.jgi-psf.org
Chlamydomonas reinhardtii	CHLRE	Plantae	Chlorophyte	3	http://genome.jgi-psf.org
Caenorhabditis elegans	CAEEL	Animalia	Nematoda	18	http://www.wormbase.org/
Escherichia coli	ESCH	Monera	Bacteria	12	http://www.ncbi.nlm.nih.gov/protein/
Saccharomyces cerevisiae (Baker’s yeast)	YGR	Fungi	Eumycota	2	http://www.yeastgenome.org/
Homo sapiens (Human)	HUMAN	Animalia	Mammalia	12	http://www.ncbi.nlm.nih.gov/genome/guide/human/

**Figure 1:**
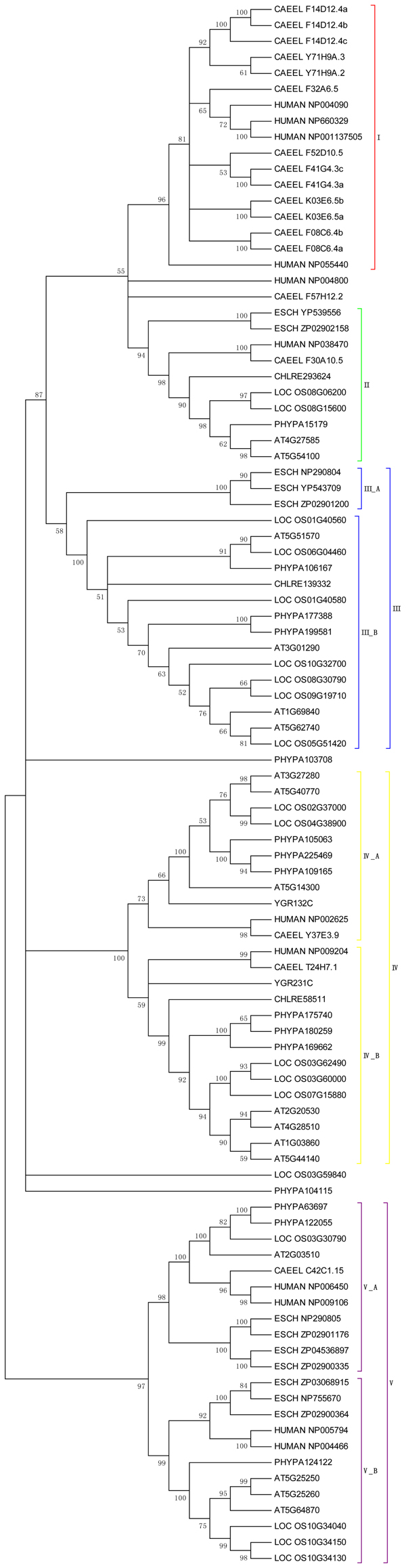
**Phylogenetic tree of PHB genes across different species.** PHB protein sequences of nine species from four kingdom were analysed, unrooted tree was constructed using Neighbour-Joining method, bootstrap 1000 replicates, only the clades with bootstrap value higher than 50 were shown. Tree was divided into five classes, some were further divided into subclasses, each class has different color indicated. Several conserved genes could not be included into classes.

We first carried out phylogenetic analysis to produce unrooted tree using the neighbor-joining method. The statistical reliability was conducted by bootstrapping 1000 replicates. The phylogenetic analysis revealed both deep evolutionary root and the existence of more recent duplications for the PHB genes. As shown in Figure 1, PHB genes from different kingdoms produced a complicated tree, where PHB genes can be classified into five groups or classes. In Class I,* C. elegans* and human PHB genes shared a clade. Both species belong to the* animalia* kingdom and the class can be considered as *animalia*-specific PHB genes. In contrary to Class I, Class II PHB genes contain the members from three different kingdoms.* Arabidopsis,* rice, *chlamydomonas,* and* Physcomitrella* (moss) are from* plantae* kingdom ranging from the higher spermatophytes to lower chlorophyte and bryophyte.* C. elegans* and human are from the* animalia* kingdom and* E.coli* is from the* monera* kingdom. The relatedness revealed the deep evolutionary origin of the Class II PHB gene, which is also confirmed by their distribution in* plantae* kingdom. The PHB genes from higher plants like* Arabidopsis* and rice shared the same clade as those from lower plant like* Physcomitrella* and* Chlamydomonas.* Class III is more of plant species-specific. However, there are also* E. coli* PHB genes. Class III thus can be divided into two subclasses; III_A for* E.coli* genes and III_B for plant genes. Class IV can also be divided into two subclasses including IV_A and IV_ B. Both subclasses have the plant genes, animal genes, and fungi (yeast) genes sharing the same clade, which indicated that Class IV PHB genes have a deep evolutionary origin too. In addition, for both subclasses, higher plant PHBs share their own clades and lower plant PHBs share distinct clades too. The results indicated that the expansion of the Class IV PHB genes in higher and lower plants is after the divergence between the higher and lower plants. Class V PHB genes also have two subclasses, where higher plant genes, lower plant genes, animal genes, and bacteria genes are separately grouped in accordance to the species evolution.

However, it is notable that* E.coli* PHB genes and human genes shared a clade together, implicating the possible horizontal gene transfer between bacteria and amniotic ancestor. The horizontally transferred PHB orthologs might have conserved molecular functions shared between eukaryotes and prokaryotes to allow the gene retention in both bacteria and human [28,29]. Generally speaking, based on the phylogenetic analysis of PHB genes across different species, the PHB gene are evolutionarily conserved and have deep evolutionary roots. In addition, it is also important to reveal the evolutionary mechanisms for more recent duplications.

**Figure 2:**
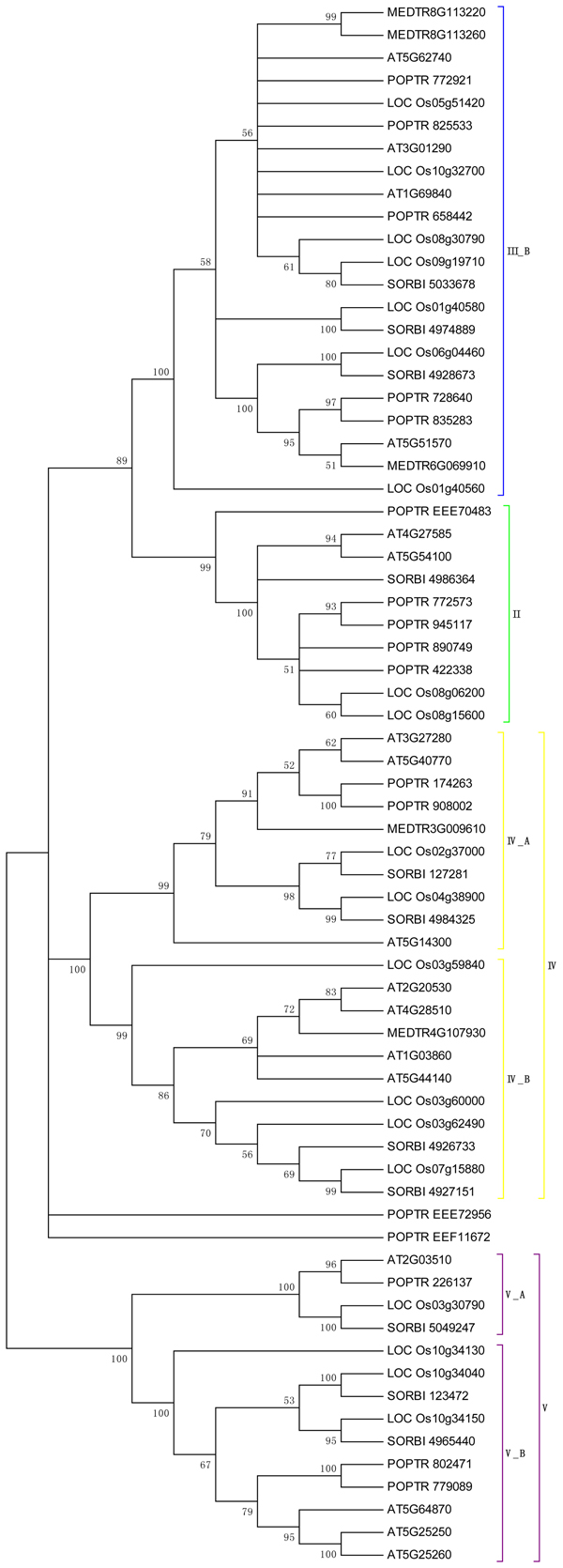
**Phylogenetic tree of PHB genes across higher species.** PHB protein sequences of four species from higher plants were analysed, unrooted tree was constructed using Neighbour-Joining method, bootstrap 1000 replicates, only the clades with bootstrap value higher than 50 were shown. The tree was classified into four classes, corresponding the Class II, III, IV, and V in the phylogenetic tree in Figure 1.

In order to further study the recent expansion of PHB genes, we focused on the relatedness of PHB genes in higher plants and chose five higher plant species including* Arabidopsis,* rice, poplar, medicago and sorghum for the further phylogenetic analysis. The un-rooted neighbor-jointing tree was built and the result is shown in Figure 2. The PHB genes in higher plants can be classified into four classes, corresponding the Class II, III, IV, and V in the phylogenetic tree in Figure 1. The phylogenetic analysis revealed the progressive evolution of the PHB genes. Generally speaking, the monocot PHB genes share clade with nearest monocot orthologs, whilst the dicot PHB genes share clade with the nearest dicot PHB genes. The results indicated the expansion of part of the gene family was after the monocot and dicot divergence at about 120 million years ago. However, if we examine the clade beyond the nearest orthologs, we can find many clades with both monocot and dicot PHB genes, which indicated that some ancestor PHB genes exist before the divergence of monocot and dicot. For example, in Class II, several poplar PHB genes shared clades with rice PHB genes, indicating the existence of an ancestor gene before the divergence of monocot and dicot species. The results correlate with the fact that Class II PHB genes have a deep evolutionary origin as indicated by Figure 1 [30].

Overall, the phylogenetic analyses revealed the seemingly contradictory phenomena, the deep evolutionary origins of the gene family and the recent expansion of the gene family. The results indicated the progressive evolution of PHB gene family because the PHB genes appears early in the evolution, but expanded at different stages during the evolution. In particular, the gene family expansion seems to continue in plant species even after the divergence between monocot and dicot, and the mechanisms for such expansion is examined in the later part of the article. The relatedness has significant functional relevance as we will discuss in the Discussion part.

### Motif analysis of PHB genes across three species

**Figure 3:**
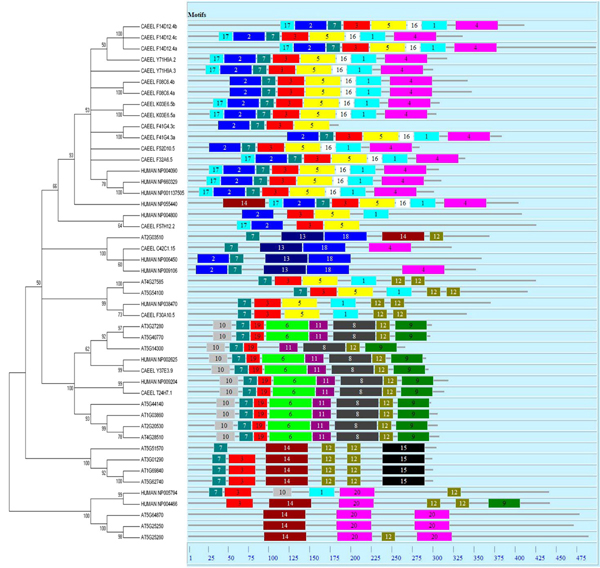
**Schematic diagram of motif distribution in Arabidopsis, human and C.elegans.** MEME 4.3.0 software was applied follow the parameters described in Method. Twenty conserved motifs were shaded in different colors. Several subgroups were distinguished by the motif distribution, which is consistant with the phylogenetic subgroups in all the three species.

The evolution of gene structure generally correlated with the phylogenetic analysis of PHB genes. We carried out three types of gene structure analyses, the motif finding, multiple sequence alignment for domain identification, and the intron/exon analysis. For the motif analysis, we chose three representative species for the study to correlate the gene structure with evolution, and these three species include human,* Arabidopsis* and* C. elegans.* The overlay of phylogenetic analysis and motif analysis is as shown in Figure 3. About twenty different subdomains or motifs between 6 to 50 residues were detected by MEME 4.3.0 software [31,32]. A clear correlation between the motif pattern and the phylogenetic analysis can be found, where each class or subclass essentially shared the same motif pattern. Some motifs like motif 7 and motif 3 are more conserved and appeared in many classes of PHB genes. These motifs could be essential elements determining the PHB domain’s common molecular function among different family members to a certain extent. Some other motifs like 6, 14, and 20 are more specific to one class or subclass of PHB genes and they might determine some specific functions of these genes[32,33]. The motif distribution indicated that the genes containing the same motifs usually produced from gene expansion within the same class or subclass no matter in higher or lower species. In other words, the ancestor genes with various motif structure seem to appear early in the evolution, and such structure has been maintained through the evolution. The motif distribution thus confirms that the PHB genes are conserved during evolution. The differences of motif distribution in different classes and subclasses of PHB genes are the structure basis for the diversity in gene functions.

### Multiple sequence alignment of PHB genes

Besides the motif finding, multiple sequence alignment is another approach to identify the conserved domain for gene function. We focused on the model species *Arabidopsis* in the multiple sequence alignment. As a model plant with the whole genome sequence available,* Arabidopsis* gene functions were widely studied. Limited research has been carried out to characterize the PHB gene in plants, and* Arabidopsis* will serve as a good model species for plant PHB gene function studies. We therefore will focus the rest of the study in* Arabidopsis.*

**Figure 4:**
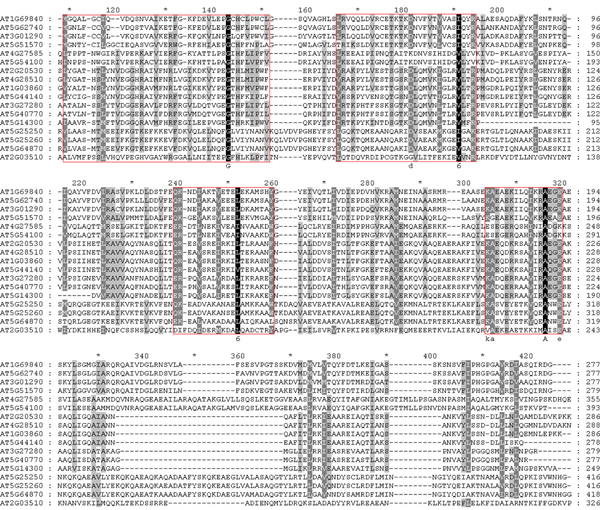
**Multiple sequence alignment of PHB proteins in Arabidopsis.** ClustalX 1.83 was applied to do complete alignment of full-length PHB proteins. Conserved residues were shaded in the conservation mode of GeneDoc software, Degree of the colors stand for different levels of conservation of each column in the alignment. Four basically conserved residue areas were cycled by red rectangles.

After sequence retrieval from TAIR database, 17* Arabidopsis* PHB proteins were collected and analyzed. ClustalX (1.83) software was used for complete multiple alignment and GeneDoc software was used for presentation as shown in Figure 4[34,35]. Across the whole sequence of PHB proteins, several conserved domains were found across all PHB genes. Basically, there were four primarily conserved regions with high similarity as colored black. In addition, several secondary and tertiary levels of conservations were detected and colored with gray and silver. It is noticeable that PHB domain spreads across a wide range of protein structure and covers many amino acid residues throughout the whole protein. Although the PHB domain was conserved in the evolutionary process from the phylogenetic analysis, it did not contain a defined region of amino acid residues that decides main functions of the protein. The phenomena is in contrary to many other gene families such as the TIFY domain of JAZ family, CCCH zinc finger domain of CCCH zinc finger family, or the ERF/AP2 domain in ERF family [36-40]. The multiple sequence alignment correlated with the motif finding, where quite diverse motif structures have been found for genes from different classes or subclasses. Combining the results from sequence alignment and motif finding, PHB genes were conserved in the existence of certain specific motifs but had rather diverse motif distributions across different classes. The structural diversity could account for the different biological functions of these genes.

### Intron/exon structure of PHB genes

**Figure 5:**
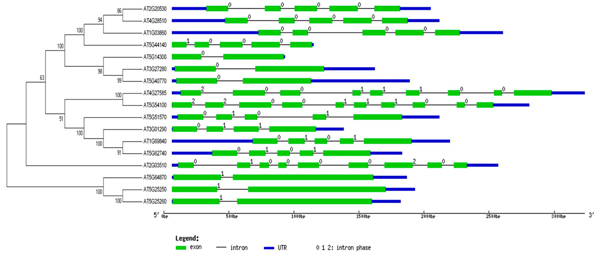
**Intron/exon structures in conjunction with phylogenetic subfamilies of PHB genes in Arabidopsis.** Gene structures were drew using online tool GSDS.As shown in the legend, green boxes stand for exons, black lines stand for introns, blue boxes are UTR regions, numbers at the exon-intron joints are intron phases. Structures of PHB genes are consistent with phylogenetic subfamily relationships.

The intron/exon structure of PHB genes were different for each class and can be divided into several groups as shown in Figure 5. We actually carried out the intron/exon structure analysis for* Arabidopsis,* human and* C. elegans.* However, the size of the intron across the species makes it misleading to present the three species together. We therefore focused on* Arabidopsis.* It is notable that the intron/exon structure correlated with the classification of PHB genes based on the phylogenetic analysis. The numbers of introns were quite different across the whole family ranging from one intron to nine introns in one gene. For example, the Class IV_A genes *At3g27280, At5g40700, At5g14300* generally have one intron; while in Class, *At2g20530, At4g28510, At1g03860, At5g44140* all have four introns. The clear correlation between intron/exon structures and the classes of PHB genes is probably due to the recent expansion of PHB genes in each subclass of PHB genes. On the other side, the PHB gene intron/exon structure thus has certain level of stability during the evolution. Intron gain/loss has played a role in the early stage evolution of PHB genes according to Figure 5. The intron/exon structure also correlates with the motif structure, where distinct patterns can be found in each subclass of PHB gene family. It is therefore expected that the gene birth due to the intron insertion or intron loss happened earlier during the evolution. The similar intron/exon structure within the subclass generally reflects the more recent gene duplications.

Both the gene length and intron phase correlate with the gene family classification and intron numbers to a certain degree. Intron phase 0, 1, and 2 referred to the splicing occurred after the first, second, and third nucleotide of the codon, respectively. As shown in Figure 5, genes with similar intron/exon structures and gene length also had conserved splicing phase patterns [41,42]. A comparative analysis of human and* C. elegans* intron/exon structure revealed much more introns in these two species as shown in Additional File 1 and 2. The results indicated that the PHB genes in* Arabidopsis* may have experienced fewer intron birth events as compared to the species in* animae* kingdom. Overall, the motif distribution, intron/exon structure, and the conserved domain all correlate well with the phylogenetic analysis and relatedness of the genes [38,40,43].

### Duplications of PHB genes in Arabidopsis

**Figure 6:**
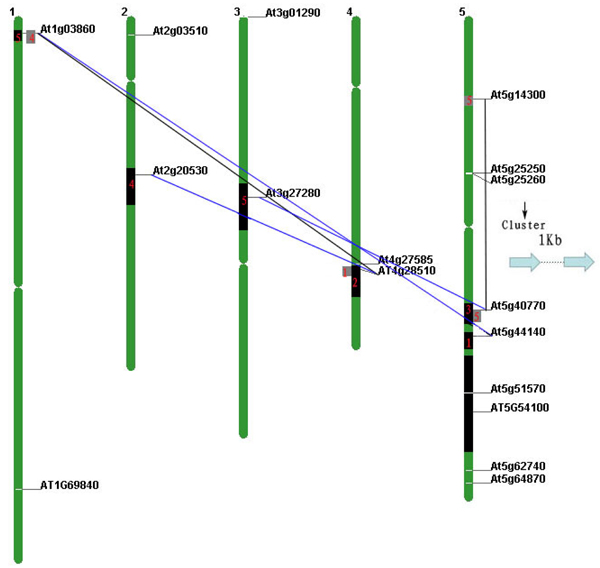
**Chromosome distribution and duplications of PHB genes in Arabidopsis.** Diagram of five chromosomes of Arabidopsis was depicted, 17 PHB family genes were distributed on the chromosomes. Only segmental duplicated regions containing PHB genes are shown. Blue lines connected genes from recent polyploidy duplication blocks (Black boxes), black lines connected genes from old segmental duplication blocks (Gray boxes).* At5g25259* and* At5g25269* are clustered as tandem repeat.

As aforementioned, recent duplication events defined the gene structure and relatedness to a certain degree, and it is important to study the duplication mechanisms to interpret the relatedness and gene structure information. There were at least two large-scale segmental duplication events in the evolutionary process of *Arabidopsis.* One was the recent polyploidy duplication, which occurred before *Arabidopsis/Brassica rapa* split around 24-40 Mya. The other was an older duplication between chromosomal blocks after the divergence of monocot-dicot around 120 Mya [44-46]. Considering these factors, we investigated PHB family gene duplication and distribution on all five* Arabidopsis* chromosomes. The recent segmental polyploidy duplicated blocks were explored by the “Paralogons in *Arabidopsis thaliana*” search engine [40]. As shown in Figure 6, there were three pairs of recent duplicated blocks containing PHB genes. The region on chromosome 1 containing* At1g03860* and the region on chromosome 5 containing* At5g44140* are duplicated segmental block pairs. The region containing* At2g20530* on chromosome 2 and the region containing* At4g28510* on chromosome 4 are duplicated segmental block pairs. The region containing* At3g27280* on chromosome 3 and the region containing* At5g40770* on chromosome 5 are duplicated segmental block pairs. All of these segmentally duplicated genes were found to be paralogs in the phylogenetic analysis as shown in Figure 1 and 2. The results indicated segmental duplication as a major way for gene birth within each class for* Arabidopsis.* However, it should also point out that not all segmental regions containing duplicated PHB genes. For example, there was a big recently duplicated block containing* At5g54100* (excluding *At5g51570*) at the bottom of chromosome 5, and its duplicated region on chromosome 4 contained no PHB genes. The results indicated that most of the segmentally duplicated PHB genes can be retained during the evolution, but some duplicated genes may have disappeared during the evolution. Besides the recent segmental duplications, there were two ancient duplication blocks also overlap with the recent segmental duplication blocks [44]. In particular, one of the ancient duplication segments on chromosome 5 was known as the so-called intra-segmental duplications and the duplicated regions contain* At5g14300* and* At5g40770,* which are paralogs in the phylogenetic analysis in Figure 2. There was not much bias of periods from inter-segmental duplications [47]. Overall, the segmental duplications play significant roles in the evolution of gene expansion, which confirms that PHB gene family is relatively conserved.

Besides the segmental duplications, one tandem duplication event was also found on chromosome 5.* At5g25250* and* At5g25260* were two genes with high similarity of DNA sequence and only 1Kb distance on the chromosome. It is very likely that the gene duplication is due to gene jumping mediated by a transposon [48]. Overall, the gene duplication pattern indicated that segmental duplication is predominant for the PHB genes and tandem duplication is also involved. The gene duplication pattern correlates very well with the relatedness of the gene.

### Expression patterns of PHB genes in* Arabidopsis*

As aforementioned, PHB genes may be involved in diverse gene functions. In order to better understand the PHB gene functions and their relevance to gene evolution, we investigated the gene expression level of PHB genes with* Arabidopsis* as the model species. The gene expression analysis included both the digital gene expression pattern using Genevestigator and the actual real-time PCR experiments.

**Figure 7:**
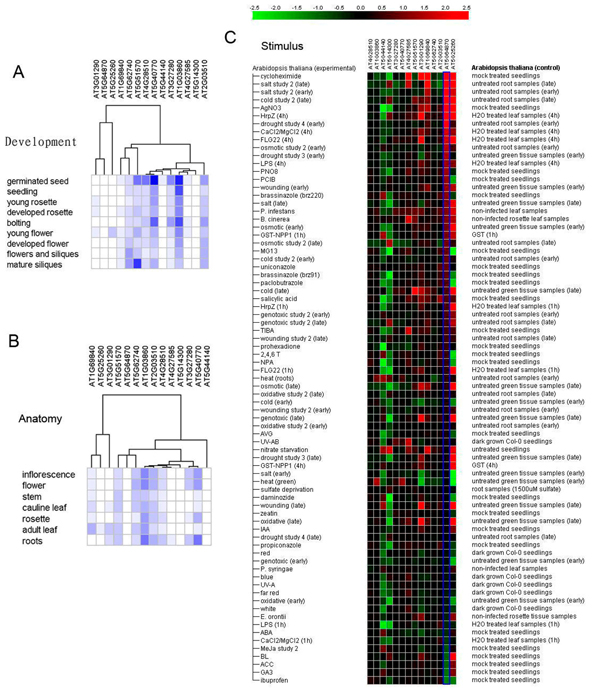
**Digital expression pattern analysis of PHB genes in Arabidopsis.** Base on public microarray data, digital expression pattern was performed by Genevestigator. Arabidopsis PHB family genes expression patterns at different development stages, in anatomical tissues and under different stimulus are shown. Hierarchical clustering was played in data analysis as shown in figure A, B.

Arabidopsis PHB family genes expression pattern at different development stages, in different tissues, and under different stimulus were analysed using Genevestigator version 3 [49]. The data from* Arabidopsis thaliana* high quality ATH1:22k microarray in the AtGenExpress was chosen to do the analysis. Developmental stage and tissue-specific expression data were analysed by hierarchical clustering as shown in Figure 7A and 7B , whilst gene expression patterns under stimulus conditions were shown using meta-profile analysis in Figure 7C [50,51].

Nine development stages were surveyed for the digital gene expression analysis. Generally speaking, PHB genes show significant variations for gene expression in terms of both the expression levels and presence at different conditions (Figure 7A). In addition, no significant gene expression pattern and phylogenetic analysis correlations were found. For example,* At1g03860* and* At4g28510/ At2g20530* could be paralogs, but they had very different expression patterns. According to the traditional gene fate evolution models, paralogs in a gene family usually have divergent expression patterns, indicating the different biological functions. Because a high gene dosage is often detrimental to the organisms, one of the paralogs often evolves a new function in a process called neofunctionalization or disappears in the evolution [52,53]. In terms of tissue-specific expression, we found that the paralog genes often have differential gene expression patterns, too (Figure 7B). The PHB genes are also responding to the biotic and abiotic stimulus treatment quite differently (Figure 7C). For example,* At5g64870* is highly up-regulated when treated with abiotic stresses such as salt, cold, drought, whilst it is down-regulated under some hormones like ABA (abscisic acid), MeJA (Methyl Jasmonate), GA (gibberellins) and so on. However, other PHB genes did not have similar expression pattern under these treatments.

**Figure 8:**
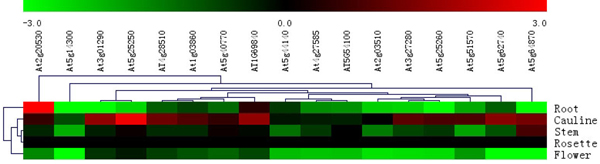
**Tissue specific Quantitative real-time PCR analysis of PHB genes in of Arabidopsis.** PHB genes expression pattern in five different tissues of Arabidopsis. Genes expressed in rosette leaf were set as control, log2 ratio of relative expression signal was used in MeV4.5.1 software. Red color represents higher expression levels, green color represent lower expression levels. Hierarchical clustering was played in data analysis.

In order to further confirm the digital gene expression pattern analysis, we carried out quantitative real time PCR experiments to analyze the tissue-specific PHB genes expression patterns as shown in Figure 8. Five* Arabidopsis* tissues of root, stem, cauline leaf, rosette leaf and flower were used. Real time PCR results also showed differential expression patterns of PHB genes. The majority of the PHB genes expression patterns were consistent with the microarray-based expression analysis.

Overall, the gene expression pattern indicated that PHB genes are involved in diverse biological functions and most of the PHB genes evolve new functions after the gene duplication, which is in contrary to some of the fast expanding gene families like terpene synthase gene family.

## Discussion

Despite the ubiquitous presence of PHB genes in prokaryotes to eukaryotes, the function and evolution of PHB genes have not been thoroughly studied. Most studies of PHB genes focused on individual gene functional analysis in yeast, mammalian,* C. elegans* and some plants[1-4,23]. Gene family analysis has become a major approach to study the gene function, evolution, and structure. The comparative analysis of gene family across multiple species allowed us to investigate how the various functions of the gene family members were evolved and how the gene structure was relevant to function [54,55]. The basic hypothesis is that conserved genes in form of orthologs often have similar functions and structures. The gene family expansion is also relevant to the interaction with herbivore or pathogens. For example, most of the plant gene families involved in insect defense like terpene synthase, cytochrome p450(CYP), WRKY gene families experienced recent and rapid evolution, partially due to the evolutionary competition with insect for chemical defense [55-57]. The analysis of the relevance of gene structure and evolution will allow us to understand how new function of a gene family member evolved and developed. Our results highlighted that PHB genes consist of a conserved family with deep evolutionary root yet diverse biological and molecular functions. The comparative analysis elucidated the evolutionary features of the PHB gene family and helped to guide our further gene function analysis and the study of PHB gene's relevance to human diseases.

### Evolution of PHB gene family

Comprehensive and concrete evolutionary analysis of PHB gene family is lacking. In order to investigate the evolution of PHB genes and the evolution-function relationship, we carried out a comprehensive phylogenetic and motif analysis of PHB genes from representative species in different kingdoms. In addition, we focused on the model plant *Arabidopsis* for further gene structure, duplication, and expression pattern analysis. Our results highlighted several features of PHB gene evolution.

First, the phylogenetic and gene structure analysis of PHB genes indicated that PHB genes are relatively conserved across different species. Most of the PHB genes within a class or subclass shared similar motif structure across plant and animal species. The intron/exon structure and domains for the genes within the same class or subclass are also conserved. Generally speaking, the PHB genes within a class or subclass share clades following the evolutionary lineage. Second, the PHB genes have deep evolutionary origins, where some homologs can even trace back to prokaryote species. The divergence of different class or subclass of PHB genes happened very early in the evolution, and some at prokaryote stage. The deep evolutionary root and conserved evolution both indicated that the PHB genes could account for some conserved molecular functions. Third, the conserved and important function can also be reflected in the gene duplication and functional divergence. Several mechanisms are involved in PHB gene family expansion. Horizontal gene transfer was also indicated between human and prokaryote. In model plant* Arabidopsis,* some PHB genes had early expansion across species in plants, and they usually have common ancestor before the species diverge [30]. However, most of the gene family expansion was due to the segmental duplication in* Arabidopsis.* Tandem duplication thus exists but is rear. The pattern is different from some dynamic gene families like Terpene Synthase, P450 and WRKY [54,58]. The results indicated that PHB genes are not much involved in the competitive evolution for chemical defense and its regulation. In fact, most of the duplicated PHB genes evolved rather different expression pattern, indicating potentially new biological function [52,53]. The result is also different from some other gene families that different gene fates exist together [54,58]. It is generally believed that plants can be tolerant to a much higher gene dosage effect as compared to animals. The fact that most of PHB gene duplications end up with paralogs with potentially different functions indicated that PHB genes would be involved in some important biological processes.

### Function of PHB gene family

The PHB gene evolution generally reflected the family’s conserved but diverse functions. From a molecular perspective, PHB genes were reported to be involved in cell-cycle progression, iron channel regulation, receptor medicated signaling, and the control of respiratory chain in mitochondria [1,5,17,26]. From a biological perspective, PHB genes were related to aging and senescence in mammalian, yeast, and* C.elegans *[24,59,60]. More importantly, they can be associated with a variety of disease states including inflammation, obesity, and cancer [5]. However, more research still need to be carried out to provide confirmative evidence to link molecular functions to biological functions.

We explored the gene expression pattern of PHB genes in model plant *Arabidopsis* to derive the functional relevance and evolution-function relationship of PHB genes. Despite the tremendous amount of research in* Arabidopsis,* very few reports were published for the function of PHB family genes. The limited previous studies indicated that PHB genes could be involved in development, senescence, hormone signaling and stress responses [22,23,61-63]. From our expression analysis results, we found that PHB genes have very diverse expression patterns in different development stages and tissues, as well as under different stimulus. The results highlighted the potential diverse biological function of PHB genes. In particular, the evolutionary pressure kept the PHB gene motif structure and intron/exon structure conserved during the evolution within each class or subclass. However, the same evolutionary pressure also seems to force the paralogs to evolve differential regulations with potentially roles for different biological processes. Much more comprehensive work needs to be carried out to study the function of PHB genes at different levels, which will also be important for the disease-related studies.

### Comparative analysis for disease study

The functional study will help to elucidate the role of PHB genes in cancer, aging, immunity, neuron degeneration and such. It was widely recognized that PHB genes played crucial roles in various human diseases [5,12,17]. Mishra et al. has reviewed the diverse localization, function and disease association of PHB genes [5]. PHB1 is also known as B-cell-receptor-associated protein 37 (BAP 37) and the 3’-UTR region of the mRNA was shown be relevant to the breast cancer phenotype [17,27]. Despite the diverse function, studies has only been focused on how PHB1 and PHB2, the first two genes found, are relevant to diseases [5,13,14,64,65]. Our comparative analysis indicated the diverse function of the gene family and the relatively conserved gene structure. The study indicated that the molecular function of PHB genes can be much more thoroughly studied in the model species that genetic tools are more readily available than human. Because of the conserved motif pattern and potential molecular function, the studies in the model species can be readily translational to the human and mammalian studies.

## Conclusions

PHB family genes are evolutionarily conserved across multiple species in the biological kingdom from our phylogenetic analysis. Gene structure and motif distributions were consistent with the evolutionary relatedness of PHB genes in Arabidopsis. Different duplication events are involved for gene family expansion, especially the segmental duplications in Arabidopsis. Horizontal gene transfer could also be involved in the birth of new genes in higher organisms. Even though PHB genes are important for a core group of molecular functions and are conserved during evolution, the members of the gene family have evolved to have very diverse biological functions in development and biotic or abiotic stress responses.

## Materials and methods

### Sequence retrieval and gene family member identification

Protein sequences were first acquired from http://www.ebi.ac.uk/interpro/ under the accession PR001107 Band_7. All sequences downloaded were searched against species specific databases with BLASTP algorism using default parameters. Redundant sequences with different accession numbers in EMBL-EBI yet the same locus id in their specific database were discarded. For example, Arabidopsis protein sequences were retrieved from TAIR http://www.arabidopsis.org/; Rice protein sequences were retrieved from TIGR http://rice.plantbiology.msu.edu/; others data source were as shown in Table 1.

Eleven species including five spermatophytes, chlorophyte, bryophyte, nematoda, bacteria, fungi and mammalian were analyzed in this study.

### Multiple sequence alignment and phylogenetic analysis

Protein sequences from different species were selected, multiple sequence alignment was performed by ClustalX (1.83) software, and the alignment result was then imported into GeneDoc (http://www.nrbsc.org/gfx/genedoc/index.html) for further visualization.

The phylogenetic tree was built by MEGA4.0 software [34,66]. The Neighbor-Joining method was used with the following parameters: pairwise deletion of gaps/missing data; poisson correlation of model; bootstrap 1000 replicates, random seed of phylogeny test. Only clades with the bootstrap value higher than 50 were selected for the bootstrap consensus tree [42,67].

### Intron/exon structure and motif analysis

Arabidopsis PHB gene CDS (Complementary DNA Sequence) and genomic sequences were used to derive intron/exon structure with the online tool Gene Structure Display Server (http://gsds.cbi.pku.edu.cn/chinese.php) [68]. Conserved motif structures within PHB domain for Arabidopsis genes were analyzed by MEME4.3.0 (Multiple Expectation Maximization for Motif Elicitation) with the following parameters; distribution of motif occurrences: any number of repetitions; number of different motifs: 20; minimum motif width: 6; and maximum motif width: 50 [31,32].

### Chromosomal distribution and duplication analysis

Arabidopsis PHB genes’s location on chromosome was mapped by the Chromosome Map Tool at TAIR (http://arabidopsis.org/jsp/ChromosomeMap/tool.jsp). “Paralogons in* Arabidopsis thaliana”* was used for detecting segmental duplication protein pairs in the recent and old duplication blocks on chromosomes separately, default parameters were set [40,44,45]. Only the blocks contained PHB genes were retained, and genes detected were then mapped on the chromosomes and linked to each other by lines manually.

### Digital expression pattern analysis

To investigate PHB genes expression profiling in Arabidopsis, Genevestigator V3 (https://www.genevestigator.com/gv/index.jsp) was used [49]. Public high quality AtGenExpress ATH1-22k microarray data was chosen. Meta-profile analysis and hierarchical clustering were used to study gene expression at different development stages, in anatomical tissues and under different stimulus[51].

### Plant growth, RNA extraction and real-time PCR experiments

*Arabidopsis* thaliana (Col-0) plants were grown under 12h light/ 12h dark photoperiod in a controlled environment chamber, 23 C at day time, 20 C at night. Specific tissues including root, stem, cauline leaf, rosette leaf and flower of six week old seedlings were collected, total RNA was extracted with RNeasy Plant Mini Kit (Qiagen). First strand cDNA was synthesized from 2ug RNA with SuperScript™ III Reverse Transcriptase (Invitrogen), then diluted to 2ng/ul. Primer sequences were designed by Primer Express3.0 (Additional File 3). Real-time PCR reaction was carried out with SYBR Green Master Mix (Applied Biosystems) according to the manufacture’s instruction. ABI 7900 sequence detection system was used. Data analysis was used MeV v4.5.1 followed the method of Xu et al., 2009 [54].

## Competing interests

The authors declare that they have no competing interests

## Authors' contributions

JSY designed the study, oversaw the work, and laid out the outline for manuscript. DC carried out the gene family analysis and wet lab experiments in JSY’s lab and also drafted the manuscript. WX, ZS, and JSY provided important suggestions for DC’s work. ZS revised the manuscript. JSY revised and finalized the manuscript.

## Supplementary Material

Additional File 1 Click here for file

Additional File 2 Click here for file

Additional File 3 Click here for file
