# Supplementary figures and images for: Comparative genome analysis of PHB gene family reveals deep evolutionary origins and diverse gene function
Source: BMC Bioinformatics. 2010 Oct 7;11(Suppl 6):S22. doi: 10.1186/1471-2105-11-S6-S22 (PMC3026370; doi:10.1186/1471-2105-11-S6-S22)

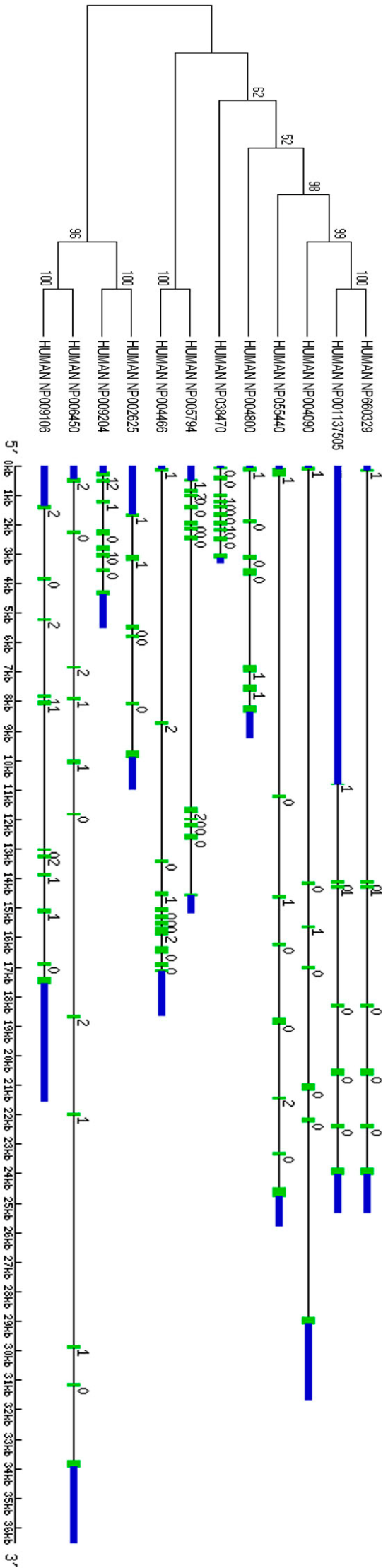

Supplement: Additional File 1 [file 1471-2105-11-S6-S22-S1.pdf]

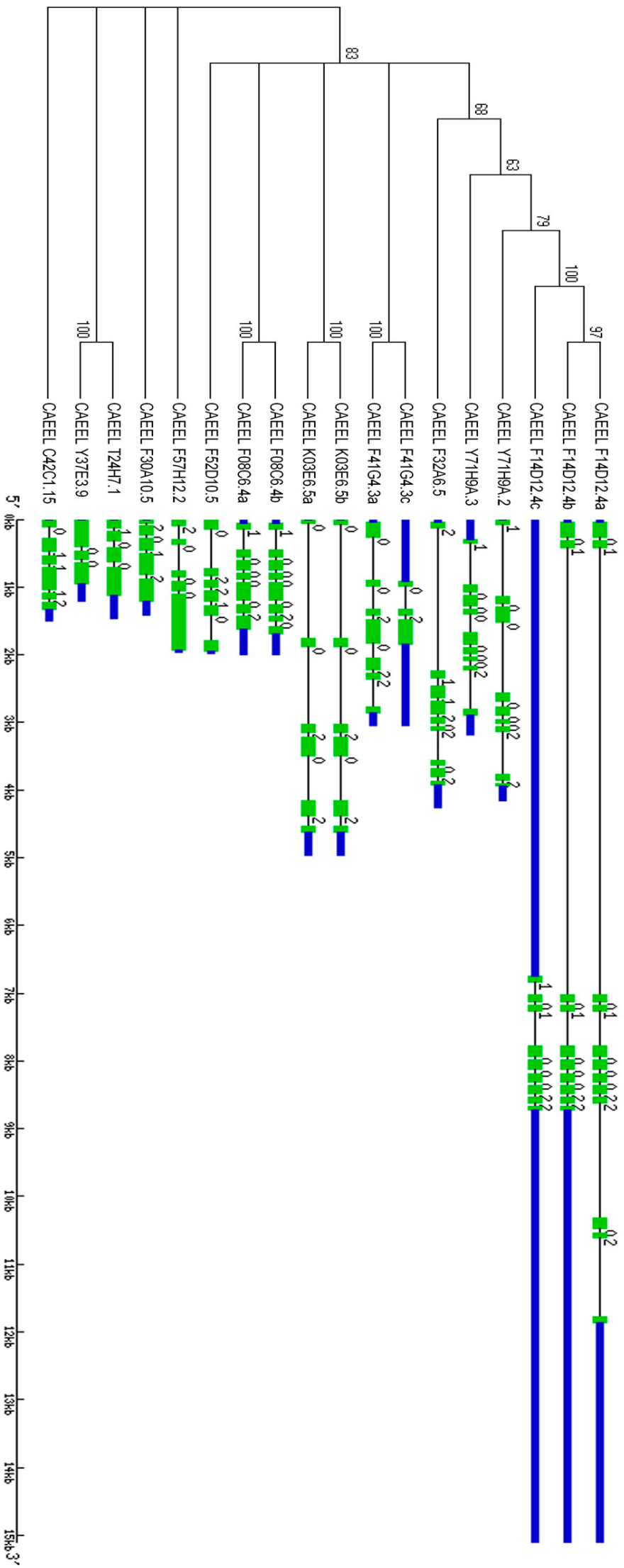

Supplement: Additional File 2 [file 1471-2105-11-S6-S22-S2.pdf]
