# Supplementary material for: Comparative genome analysis of PHB gene family reveals deep evolutionary origins and diverse gene function
Source: BMC Bioinformatics. 2010 Oct 7;11(Suppl 6):S22. doi: 10.1186/1471-2105-11-S6-S22 (PMC3026370; doi:10.1186/1471-2105-11-S6-S22)
Supplement: Additional File 3 [file 1471-2105-11-S6-S22-S3.pdf]

	primer name	sequences	pcr product length(bp)
Forward	At1g03860-F	ATTGGTGGGCTCGGTGTCT	63
Reverse	At1g03860-R	ACGGTGTCCTCCATCGACAT	
Forward	AT1G69840-F	AATGAGAGAGGCAGCGAGTGA	61
Reverse	AT1G69840-R	AGCACGCTTGATCTGCAGAAT	
Forward	At2g03510-F	CGCCGCCTTGGTTATGTT	60
Reverse	At2g03510-R	CCAACGTGACCTTCAGGAACTT	
Forward	At2g20530-F	AGGAGTTCACGGCAGCCATA	60
Reverse	At2g20530-R	TAGCCCGTTCGGCCTCTT	
Forward	At3g01290-F	CCGGTACACTGACCCTTCGT	64
Reverse	At3g01290-R	TTGTCCTTTGTTTTGGTTTCACA	
Forward	At3g27280-F	TCCGAACGTGGCTTACTTACCT	64
Reverse	At3g27280-R	TCAACGACCAGGGTTCAGATT	
Forward	At4g27585-F	CCGGAGAGGAAGGCGTTT	57
Reverse	At4g27585-R	CGGCAACGTCGTAGCGTAT	
Forward	AT4g28510-F	GGTGGGCTTGGTCTCTATGGT	60
Reverse	AT4g28510-R	TCGATGTCCTCCTTCAACATTG	
Forward	At5g14300-F	CTCAGCTCTTATACGCGAAACG	65
Reverse	At5g14300-R	CGTCATCGAGAACAATGTTGAAC	
Forward	At5g25250-F	CGGTGCTGGTATCGAAGACAT	66
Reverse	At5g25250-R	GTGCAAGATTGCCATGGAAA	
Forward	At5g25260-F	GCAGCCCAAGATTAGCGTTT	61
Reverse	At5g25260-R	CCCACTAGCACCACCAATCC	
Forward	At5g40770-F	TTCAAGATAATCCACACGCAAAA	66
Reverse	At5g40770-R	CCAAATTGGAAAGGAACGAAAC	
Forward	At5g44140-F	ACAAGTTGCGGCTCAGGAA	63
Reverse	At5g44140-R	TTGTCTTGCTCGGCCTTTTC	
Forward	At5g51570-F	CTCCAGCTCGCGAGTGTTTAC	61
Reverse	At5g51570-R	CGGCTCTCTTCACTTGGAGAA	
Forward	AT5G54100-F	CGCTTTCAAGATCCGGTTACA	63
Reverse	AT5G54100-R	GAACAGCTGCGGAAAGCATAG	
Forward	At5g62740-F	CAGGCGATTGTCGATGGAT	58
Reverse	At5g62740-R	CAGGGACATTCACAGCGAAAC	
Forward	At5g64870-F	GGCAGTCTTGCACCGTCTTC	64
Reverse	At5g64870-R	CTCATAGCTTGAACCTCGAACGT	
